# Analyzing 2D gel images using a two-component empirical bayes model

**DOI:** 10.1186/1471-2105-12-433

**Published:** 2011-11-08

**Authors:** Feng Li, Françoise Seillier-Moiseiwitsch

**Affiliations:** 1 Department of Mathematics and Statistics, University of Maryland, Baltimore County, Baltimore, Maryland, USA; 2 Division of Biometrics II, Office of Biostatistics, Center for Drug Evaluation and Research, Food and Drug Administration, 10903 New Hampshire Avenue, Silver Spring, MD 20993, USA; 3 Infectious Disease Clinical Research Program, Department of Preventive Medicine and Biometrics, Uniformed Services University of the Health Sciences, Bethesda, Maryland, USA

## Abstract

**Background:**

Two-dimensional polyacrylomide gel electrophoresis (2D gel, 2D PAGE, 2-DE) is a powerful tool for analyzing the proteome of a organism. Differential analysis of 2D gel images aims at finding proteins that change under different conditions, which leads to large-scale hypothesis testing as in microarray data analysis. Two-component empirical Bayes (EB) models have been widely discussed for large-scale hypothesis testing and applied in the context of genomic data. They have not been implemented for the differential analysis of 2D gel data. In the literature, the mixture and null densities of the test statistics are estimated separately. The estimation of the mixture density does not take into account assumptions about the null density. Thus, there is no guarantee that the estimated null component will be no greater than the mixture density as it should be.

**Results:**

We present an implementation of a two-component EB model for the analysis of 2D gel images. In contrast to the published estimation method, we propose to estimate the mixture and null densities simultaneously using a constrained estimation approach, which relies on an iteratively re-weighted least-squares algorithm. The assumption about the null density is naturally taken into account in the estimation of the mixture density. This strategy is illustrated using a set of 2D gel images from a factorial experiment. The proposed approach is validated using a set of simulated gels.

**Conclusions:**

The two-component EB model is a very useful for large-scale hypothesis testing. In proteomic analysis, the theoretical null density is often not appropriate. We demonstrate how to implement a two-component EB model for analyzing a set of 2D gel images. We show that it is necessary to estimate the mixture density and empirical null component simultaneously. The proposed constrained estimation method always yields valid estimates and more stable results. The proposed estimation approach proposed can be applied to other contexts where large-scale hypothesis testing occurs.

## Background

Complementing functional genomics, proteomics deals with the large-scale analysis of proteins expressed by a tissue under specific physiological conditions. A broad range of technologies are used in proteomics, but the central paradigm has been the use of a method for separating mixtures of proteins followed by identification of protein by mass spectrometry (MS). Two-dimensional polyacrylomide gel electrophoresis (2D PAGE, 2D gel, 2-DE) very popular, despite the availability of other powerful separation techniques. With 2D PAGE [1], proteins are separated in one dimension according to their molecular mass and in the orthogonal dimension according to their isoelectric charge. In theory, each protein is uniquely determined by its location along the two dimensions of separation. The separated proteins are then stained with fluorescent dyes so that they are amenable to imaging. Proteomic differences across multiple samples can be studied by comparing the expression profiles across sets of gels.

**Figure 1:**
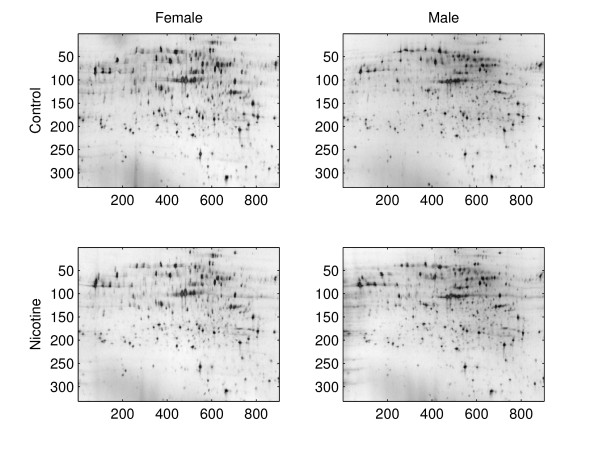
**Images of proteomes from rat spleens**.

Figure 1 shows typical images of 2D gels. Each dark spot with a smooth contour represents a different protein. The darkness of a spot is proportional to the quantity of the corresponding protein on the gel. By comparing spot intensities across images, we are able to compare the volumes of the same protein under different treatments or exposures or stages of tissue development and identify protein spots that change in volume under conditions of interest. It would be unwieldy to do this manually since there are thousands of spots to compare and gels undergo distortions during the experimental process.

The main steps in differential analysis of two-dimensional gels involve image de-noising, spot detection, spot quantification, spot matching and statistical analysis, which were discussed in detail in [2]. Unlike the analysis of microarray data, the statistical differential analysis of 2D gel images is still in its infancy. The main difficulties are the discrimination between actual protein spots and noise, the quantification of protein expression levels thereafter, and spot matching for individual comparison. Although there are commercial software packages for 2D gel image analysis (e.g. PDQuest, Dymension), considerable human intervention is required for spot matching. Spot matching is the process by which one maps a spot on a particular gel to the corresponding spots on the other gels so that spots corresponding to the same protein are identified. With a larger number of images, this step becomes increasingly problematic as fewer spots are matched and the analysis is performed on sparser data [3]. Moreover, in available software packages, the comparison of the quantitative features is based on classical tests, such as the t-test or the F-test. Attempts have been made to avoid image segmentation and spot quantification. Models based on image pixels [4] are not practical given the huge number of pixels, high variation in the background intensity and sensitivity to misalignment.

Recently, academic software was developed to cope with difficulties in the analysis pipeline including protein spot detection, quantification and spot matching [3,5,6]. To improve the spot-detection results and avoid spot matching, the methods in [3,6] utilize the mean gel image as the template for locating spots. The pinnacle method [3] uses a fixed window for spot detection, quantification and background separation. The approaches in [5,6] rely on the watershed transform [7] for spot segmentation and quantification. The RegStaGel software [6] provides advanced statistical tools. Comparison of different software for protein spot quantification is beyond the scope of the current paper. We shall focus on the statistical analysis, assuming that spot quantification has been performed appropriately. For convenience, we employ RegStatGel [6] to obtain spot quantification for statistical analysis of the set of gel images considered in this paper.

Since hundreds or thousands of proteins are usually featured on a gel, once proteins are quantified, we are faced with a large-scale hypothesis-testing problem. The RegStatGel software [6] applies the Benjamini-Hochberg (BH-FDR) procedure [8] in combination with multivariate analysis for identifying significantly changed proteins. The BH-FDR procedure is widely used for selecting the p-value threshold to control the false discovery rate (FDR). Under the assumptions that tests are independent or weakly dependent and the null distribution of the p-values is uniform, the BH-FDR procedure controls the false-discovery rate at a given level. But in practice, these two assumptions are often invalid. Strong dependence usually exists, especially in the field of genomics and proteomics [9], where the dependencies themselves are actually also of interest. Considerable effort has been dedicated to the estimation of the proportion of true null hypotheses and of the false discovery rate at a given p-value threshold [10-19]. The empirical Bayes methodology and closely related methods exploiting a two-component mixture model [10,15,20,21] represent typical examples of such effort. The two-component EB models assumes that a test statistic follows either the null or the non-null distribution.

It has been commonly assumed that the null distribution of the test statistics follows some distribution theoretically. However, Efron [12-15] pointed out that in large-scale hypothesis testing the theoretical null distribution often does not hold for reasons including incorrect model assumption, unobserved covariates and correlations among test statistics. It is more appropriate to estimate the null density of the test statistics directly from the data instead of using the theoretical null density. Using the two-component empirical Bayes (EB) model, Efron [12-15] proposed to estimate the mixture density from the entire histogram and the null component from data around the central peak of the mixture density. The two-component EB model aims at separating a small subset interesting cases from a large group of uninteresting cases. Efron's innovative concept and estimation approach have been throughly discussed [22-26]. The *locfdr *R package [27] was developed to estimate the two-component model using Poisson regression and computing the local false discovery rate (FDR).

Two methods [12,15] were proposed to estimate the null component. One is based on finding an optimal normal approximation to the mixture density around the central peak of the histogram, and the other on maximum-likelihood estimation. In both methods, the mixture density and the null component are estimated separately. The estimation of the mixture density does not take assumptions about the null density into account. Thus, there is no guarantee that the estimated null component is no greater than the mixture density over the entire domain. The two approaches may result in the estimated local FDR having multiple peaks or its being greater than 1 [25]; neither is desirable. We present a modified estimation method for the two-component EB model: the null and the mixture densities are estimated simultaneously with a necessary constraint, which can be achieved with a constrained iteratively re-weighted least squares (IRLS) algorithm. The proposed methodology is applied to the analysis of a set of 2D gel images from a factorial experiment. Simulation studies are conducted to further validate and investigate the performance of the proposed approach.

## Methods

### Data

To investigate the effect of nicotine exposure on the proteome of spleen cells of female and male rats, a 2 × 2 factorial design with gender and treatment (nicotine exposure) factors was used with 3 rats in each experimental group. Spleen cells from the control and treated rats were harvested on post-natal day 65 and then cultured in the presence of convanavalin A. After 4 days in culture, cell pellets were lysed and solubilized directly in rehydration buffer. Lysates were aliquoted and stored frozen at -80°C. Samples were thawed and 20 *μ*g protein from each sample applied to a pH 4-7 immobilized pH gradient strip (IPG; Amersham Biosciences/GE Healthcare) by overnight rehydration. Isoelectric focusing was performed using an IPGphor IEF system (Amershan Biosciences/GE Healthcare) with voltage increased gradually from 500 to 800 V and then kept constant at 8000 volts for 4 hours. Separation in the second dimension was performed on 12.5% Excel prepared gels specifically made for the Multiphor II apparatus (Amersham Biosciences/GE Healthcare) and run at 40 mA for 35 minutes followed by 100 mA for 1.25 hours. Gels were silver stained (Amersham Plus One silver stain kit) and imaged using a UMACS Power Look 3 scanner (Amersham).

Figure 1 shows four images, each from a different experimental group. The top row has examples of control rats and the bottom row of rats exposed to nicotine. The left column has examples for female rats and the right column of male rats. First, the images were aligned using the algorithm described in [28]. After alignment, boundaries for the interesting portion of the images were set and the region outside these boundaries was cropped.

The objective is to find proteins that changed in quantity under exposure to nicotine or show a gender effect. The next steps would be to determine the genomic sequence of the differrentially-expressed proteins by mass spectrometry and to refer these sequences to a database of protein sequences in order to identify them and investigate their functions.

The proteins were detected and quantified using the the default settings of the RegStatGel software [6,29]. Specifically, the watershed algorithm was applied to the mean image to generate a master watershed map which is then imposed onto each individual gel image. Each watershed region contains a single object, either a single spot or an aggregate of two spots 9a seldom occurence). The pixels in each region are then classified as either belonging to the object or to the background using Otsu's method [30]. The mean intensity difference between the object and background serves as a summary statistic for each region and therefore for each protein (or aggregate), and is used for comparison across images. The RegStaGel software is fast, easy to use and has comparable performance to commercial software packages [29]. Note that other free programs such as Pinnacle [3] can also be used for protein quantification.

For the dataset under consideration, there are a total of 439 watershed regions containing proteins (including overlapping spots). Now, we set up a statistical model for comparing protein quantities across experimental groups. Denote the log of the statistic of interest (e.g. average pixel intensity, total pixel intensity, mean intensity difference) for protein *i*, image *l*, experimental group *g *by *y_gli_*, where *g *= 1, ..., *n_c_*, *l *= 1, ..., *n*, *i *= 1, ..., *K*. For the dataset described above, we have *n_c _*= 4, *n *= 3, *K *= 439, and the experimental conditions (*g *= 1, ..., 4) correspond to the factorial combinations of treatment and gender. We have the following linear model:

(1) $$\begin{array}{c} y_{1li}=\mu_{i}-\tau_{i}-\gamma_{i}-{\left(\tau \gamma \right)}_{i}+\varepsilon_{1li}\\ y_{2li}=\mu_{i}-\tau_{i}+\gamma_{i}+{\left(\tau \gamma \right)}_{i}+\varepsilon_{2li}\\ y_{3li}=\mu_{i}+\tau_{i}-\gamma_{i}+{\left(\tau \gamma \right)}_{i}+\varepsilon_{3li}\\ y_{4li}=\mu_{i}+\tau_{i}+\gamma_{i}-{\left(\tau \gamma \right)}_{i}+\varepsilon_{4li} \end{array}$$

where *τ_i_*, *γ_i_*, (*τγ*)_*i *_are, respectively, the treatment, gender, and interaction effect for protein *i*. With the assumption that $\varepsilon_{gli}\sim N\left(0,\;\sigma_{i}^{2}\right)$, the test statistic for the treatment effect on protein *i *is

$$t_{i}=\frac{{ȳ}_{3.i}+{ȳ}_{4.i}-{ȳ}_{1.i}-{ȳ}_{2.i}}{2\sqrt{S_{i}∕n}},$$

where *S_i _*is the pooled sample variance and *t_i _*follows the *t*-distribution with *df *= 4(*n *- 1) degrees of freedom under the null hypothesis that *τ_i _*= 0. The test statistics for the gender and interaction effects follow the same *t*-distribution under the null hypothesis. Let *z_i _*= Φ^-1^(*F_df_*(*t_i_*)), where *F_df _*is the cumulative *t_df _*distribution. Theoretically, under the null hypothesis, *z_i _*follows the standard normal distribution.

### Two-component Empirical Bayes Model

The two-component EB model assumes a mixture model for the density of *z_i_*,

$$f\left(z_{i}\right)=p_{0}f_{0}\left(z_{i}\right)+\left(1-p_{0}\right)f_{1}\left(z_{i}\right),$$

where *p*_0 _is the prior probability that *z_i _*complies with the true null hypothesis, *f*_0_(*z_i_*), is the null density and *f*_1_(*z_i_*) is the density under the alternative hypothesis. This model is very popular in the literature on differential analysis of microarray data, where most authors assume the null density is the theoretical null density.

Efron [10,15] defined the posterior probability that *z_i _*is from the null hypothesis as the local FDR, which is given by

$$fdr\left(z_{i}\right)=Pr\left(H_{0i}\;\text{is true}|Z=z_{i}\right)=p_{0}f_{0}\left(z_{i}\right)∕f\left(z_{i}\right).$$

It can be shown [12,15] that the relationship of the local FDR to the usual FDR is

$$FDR\left(z_{i}\right)=E_{f}\left\{fdr\left(Z\right)|Z\leq z_{i}\right\}.$$

To estimate the local FDR, we must estimate the unknown *p*_0_, *f*_0_, *f*. Theoretically, *f*_0 _should be the *N*(0, 1) density. However, for many reasons, this theoretical null density may not be valid in practice. For example, strong correlations among tests or covariates unaccounted for in the model will invalidate the usual assumptions [12-15]. Moreover, when the majority of tests show small effects, it is sounder to select the relatively more interesting effects by comparing larger effects to smaller effects rather than to the theoretical zero effects. Therefore, it is more appropriate to estimate the null density of the test statistics directly from the data instead of using the theoretical null distribution.

Efron [12,15] assumed the null distribution to be *N*(*δ*, *σ*^2^) and estimated the null distribution from the data. The log of the mixture density log(*f*(*z*)) was estimated by fitting a natural cubic spline or high-order polynomial to the log of counts in the histogram bins via Poisson regression. Indeed, suppose the *z*-values have been binned and the bin counts are

$$m_{j}=\#\left\{z_{i}\;\text{in bin}\;j\right\},\;j=1,2,\ldots ,J.$$

Assume the *m_j_*'s to be Poisson counts, i.e.

$$m_{j}\sim P_{o}\left(\nu_{j}\right),\;j=1,\ldots ,J,$$

with the unknown *ν_j _*proportional to the density *f*(*x_j_*) at the midpoint *x_j _*of bin *j*, i.e. approximately

$$\nu_{j}=N\Delta f\left(x_{j}\right),$$

where Δ is the width of the bin and *N *is the total number of tests. log(*ν_j_*) can be modeled using a polynomial function at *x_j _*or a natural cubic spline and estimated using standard generalized linear models (GLM) for Poisson observations.

### Efron's estimation methods for the empirical null distribution

Both the central matching (CME) and the maximum likelihood (MLE) methods of estimation are implemented in the *locfdr *R package [15,27]. MLE is somewhat more stable but can be more biased than CME. Efron [12] shows that CME yields nearly unbiased estimates.

#### Central matching

When *z_i _*is generated from a t-test, the central peak of the histogram is assumed to coincide with the null density. To estimate the empirical null density from the estimated mixture density, a quadratic curve $\log\left(\hat{p_{0}f_{0}}\left(z\right)\right)$ is fitted to the central peak of $\log\left(\hat{f}\left(z\right)\right)$,

$$\log\left(\hat{p_{0}f_{0}}\left(z\right)\right)={\hat{\beta }}_{0}+{\hat{\beta }}_{1}z+{\hat{\beta }}_{2}z^{2}.$$

Assuming *f*_0_(*z*) ~ *N*(*δ*, *σ*^2^), the log of the null component is

$$\begin{array}{ccc} \log\left(p_{0}f_{0}\left(z\right)\right)= & \log p_{0}-\frac{1}{2}\left\{\frac{\delta^{2}}{\sigma^{2}}+\log\left(2\pi \sigma^{2}\right)\right\} & \\ & +\frac{\delta }{\sigma^{2}}z-\frac{1}{2\sigma^{2}}z^{2}. \end{array}$$

*p*_0_, *δ*, and *σ *can be estimated from ${\hat{\beta }}_{0},{\hat{\beta }}_{1}$, and ${\hat{\beta }}_{2}$. The local FDR at *z *is then estimated by $\hat{fdr}\left(z\right)=\hat{p_{0}f_{0}}\left(z\right)∕\hat{f}\left(z\right)$. The quadratic curve is obtained by finding a least-squares approximation to the estimated $\log\left(\hat{f}\left(z\right)\right)$ using bins in a selected interval [*a*, *b*] containing null *z_i_*'s.

#### Maximum likelihood estimation

An alternative estimation method is based on the maximum-likelihood estimator of the parameters *p*_0_, *δ*, *σ*. Assume that the non-null density *f*_1_(*z*) is supported outside some given interval [*a*, *b*]. Let *N*_0 _be the number of *z_i _*in [*a*, *b*], and define

$$P_{0}\left(\delta ,\;\sigma \right)=\Phi \left(\frac{b-\delta }{\sigma }\right)-\Phi \left(\frac{a-\delta }{\sigma }\right)\;\text{and}\;\theta =p_{0}P_{0}.$$

Then the likelihood function for all the *z*-values in [*a*, *b*] is

$$f_{\delta ,\sigma ,p_{0}}\left(z\right)\propto \left[\theta^{N_{0}}{\left(1-\theta \right)}^{N-N_{0}}\right]\left[\underset{z_{i}\in \left[a,b\right]}{\prod }\frac{\phi_{\delta ,\sigma }\left(z_{i}\right)}{P_{0}\left(\delta ,\sigma \right)}\right],$$

where *ϕ *denotes the normal density. The estimates of *p*_0_, *δ*, and *σ *can be obtained by maximizing this likelihood.

### Constrained Estimation Approach

**Figure 2:**
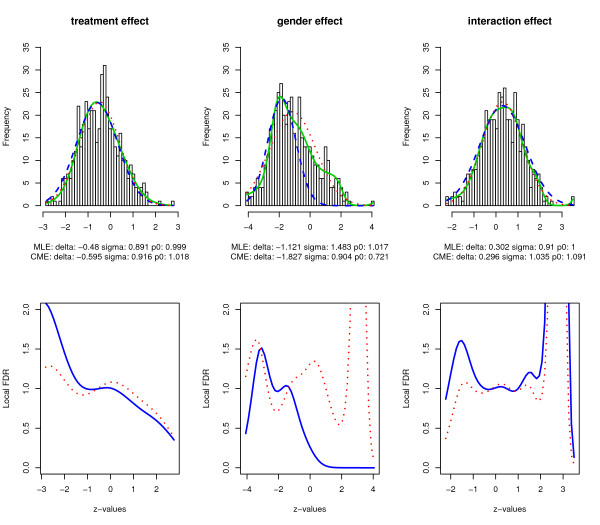
**Estimation results using unconstrained approaches**. Estimates of mixture densities and their null components from the CME and MLE methods, and the local FDR. Upper panel: solid green curves are the spline-fitted mixture densities; the blue dashed and red dotted curves are the empirical null densities from the CME and MLE methods, respectively. Lower panel: the local FDR estimates from the CME (blue solid curve) and MLE (red dotted curve) methods.

In the procedures described above, the mixture density and its null component are estimated separately. The estimated null component $\hat{p_{0}f_{0}}\left(z\right)$ may be greater than the mixture density $\hat{f}\left(z\right)$. Thus, there is no guarantee that we will have $\hat{fdr}\left(z\right)=\hat{p_{0}f_{0}}\left(z\right)∕\hat{f}\left(z\right)\leq 1$ for all *z*. Indeed, we may end up awkwardly having that $\hat{fdr}\left(z_{1}\right)>1>\hat{fdr}\left(z_{2}\right)$ for some *z*_1 _<*z*_2 _< 0, as shown in Figure 2, where both approaches were implemented on the set of gels of interest.

Therefore, we propose to modify the CME approach by estimating the mixture density and its null component simultaneously. The log of the null component is estimated via a quadratic approximation to the central peak of $\log\left(\hat{f}\left(z\right)\right)$ using bins contained in the interval [*a*, *b*]. We add the constraint that $\hat{f}\left(x_{j}\right)\geq \hat{p_{0}f_{0}}\left(x_{j}\right)$ (for all histogram bins *x_j_*) while maximizing the Poisson likelihood. To solve this problem, we utilize a constrained iteratively reweighted least-squares algorithm, as shown below. We approximate the bin counts of the mixture histogram via Poisson regression using a natural cubic spline with *D *knots. Assume the knots are *x*_1 _= *h*_1 _< ⋯ <*h_D _*≤ *x_J_*, where *x*_1 _and *x_J _*are the two bins at the left and right ends of the histogram. Denote the value of the natural cubic-spline function at point *x *by *s*(*x*; *θ*), where *θ *is the unknown parameter vector for the cubic splines. Then

$$s\left(x;\theta \right)=\overset{D}{\underset{d=1}{\sum }}B_{d}\left(x\right)\theta_{d}=B{\left(x\right)}^{\prime }\theta$$

where *θ *= [*θ*_1_, ..., *θ_D_*]', *B*(*x*) = [*B*_1_(*x*), ..., *B_D_*(*x*)]'. *B_d_*(*x*) are the natural cubic spline basis functions [31]:

$$\begin{array}{c} B_{1}\left(x\right)=1,\;B_{2}\left(x\right)=x,\\ B_{d}\left(x\right)=\phi_{d-2}\left(x\right)-\phi_{D-1}\left(x\right),\;d=3,\;\ldots ,\;D, \end{array}$$

where $\phi_{d}\left(x\right)=\left[{\left(x-h_{d}\right)}_{+}^{3}-{\left(x-h_{D}\right)}_{+}^{3}\right]∕\left(h_{D}-h_{d}\right)$ and (*x *- *h_d_*)_+ _= 0 if *x *<*h_d_*. We fit the log of the histogram counts using the natural cubic spline assuming

$$\log\left(\nu_{j}\right)=\log\left(N\Delta \right)+\log\left(f\left(x_{j}\right)\right)=s\left(x_{j};\theta \right).$$

Suppose the non-null density is close to zero in [*a*, *b*], we have approximately for *x_j _*∈ [*a*, *b*]

$$\log\left(\nu_{j}\right)\approx \log\left(N\Delta \right)+\log\left(p_{0}f_{0}\left(x_{j}\right)\right)=q\left(x_{j};\beta \right)$$

where *q*(*x*; *β*) is a quadratic function with parameter *β*.

The constraint that log (*f*(*x_j_*)) ≥ log (*p*_0 _*f*_0_(*x_j_*)) leads to *s*(*x_j_*; *θ*) ≥ *q*(*x_j_*; *β*) for all *x_j_*'s. Then, we only need to estimate the parameters *θ *by maximizing the Poisson likelihood with the constraint that *s*(*x_j_*; *θ*) ≥ *q*(*x_j_*; *β*), which results in solving

(2) $$\begin{array}{c} \max\sum L\left(m_{j},\;x_{j};\theta \right)\\ \text{subject to}\;s\left(x_{j};\theta \right)\geq q\left(x_{j};\beta \right),\;j=1,\;\ldots ,\;J \end{array}$$

where *L*(*m_j_*, *x_j_*; *θ*) = -exp{*s*(*x_j_*; *θ*)} + *m_j _s*(*x_j_*; *θ*). *L*(*m_j_*, *x_j_*;*θ*) is the Poisson log likelihood for bin *j*, omitting the constant term unrelated to the parameter *θ*. *q*(*x*; *β*) is the best quadratic approximation to *s*(*x*; *θ*) based on bins in [*a*, *b*]. To solve this, the parameter *β *must be expressed as a function of *θ*. Below, we show how to re-write the constraint in terms of the spline parameter *θ*.

Denote the values of the natural cubic spline at all the bins *x*_1_, ..., *x_J _*as a vector *S*(*θ*). We have

$$S\left(\theta \right)=\left[B{\left(x_{1}\right)}^{\prime },\cdots \;,B{\left(x_{J}\right)}^{\prime }\right]\theta =\Gamma \theta ,$$

where Γ, a *J *× *D *matrix, has entry in row *j *and column *d *Γ(*j*, *d*) = *B_d_*(*x_j_*). Similarly, we denote the values of the spline at bins in [*a*, *b*] in a vector form as *S*_0_(*θ*) = Γ_0_*θ*, where Γ_0 _is the corresponding sub-matrix of Γ. *S*_0_(*θ*) approximates the null component of the mixture density. Let *q*(*x*; *β*) = *ω*(*x*)'*β *be a quadratic function, where *ω*(*x*) = [1, *x*, *x*^2^]' and *β *= [*β*_1_, *β*_2_, *β*_3_]'. The values of the quadratic function at all bin midpoints can be written in a vector form as *Q*(*β*) = Ω*β*, where Ω is the *J *× 3 matrix with *j*th row as *ω*(*x_j_*) for *j *= 1, ..., *J*. Similarly, we denote the values of the quadratic function at bin midpoints *n *[*a*, *b*] as *Q*_0_(*β*) = Ω_0_*β*, where Ω_0 _is the submatrix of Ω corresponding to bins in [*a*, *b*].

We want to obtain the best quadratic approximation to the natural cubic spline *s*(*x*; *θ*) based on the bin midpoints *x_j _*∈ [*a*, *b*]. The least-squares solution minimizing (Γ_0_*θ *- Ω_0_*β*)'(Γ_0_*θ *- Ω_0_*β*) is given by

Thus, maximizing the likelihood (2) is equivalent to solving

(3) $$\begin{array}{c} \max\sum L\left(m_{j},\;x_{j};\theta \right)\\ \text{subject to}\;\left(\Gamma -\Omega {\left({\Omega^{\prime }}_{0}\Omega_{0}\right)}^{-1}{\Omega^{\prime }}_{0}\Gamma_{0}\right)\theta \geq 0. \end{array}$$

The above problem is solved by means of non-linear programming. A simple computational algorithm for estimating the null and mixture densities is to modify the iteratively reweighted least-squares (IRLS) procedure [32] for Poisson regression by adding the constraint to the weighted least-squares regression. The IRLS algorithm converges very fast, based on our experience.

The pseudo code for the modified IRLS algorithm is as follows:

/* Initialization of deviance Dev and oldDev */

Dev = 100000, oldDev = 0

/* Initialization of estimation of ν_k _*/

$\nu_{j}=\left(m_{j}+\frac{1}{J}\sum m_{j}\right)∕2$

Where (|Dev-oldDev| > tolerance)

{

/* Update weights */

*w_j _*= *ν_j_*

${\tilde{m}}_{j}=\log\left(\nu_{j}\right)+\left(m_{j}-\nu_{j}\right)∕\nu_{j}$

/* Constrained weighted regression */

$\begin{array}{cc} \theta =\arg\min & \sum w_{j}{\left(s\left(x_{j};\theta \right)-{\tilde{m}}_{j}\right)}^{2}\\ & \text{s.t. }\left(\Gamma -\Omega {\left(\Omega_{0}^{\prime }\Omega_{0}\right)}^{-1}\Omega_{0}^{\prime }\Gamma_{0}\right)\theta \geq 0 \end{array}$

*ν_j _*= exp{*s*(*x_j_*; *θ*)}

/* Update Poisson deviance */

oldDev = Dev

Dev = 2Σ{*m_j _*log(*m_j_*) - *m_j _*- (*m_j _*log(*ν_j_*) - *ν_j_*)}

}

The local FDR can then be estimated using $\hat{fdr}\left(z\right)=\exp\left\{q\left(z;\hat{\beta }\right)-s\left(z;\hat{\theta }\right)\right\}$, where .

## Results and Discussion

In this section, we implement the two-component EB model on the set of 2D gel images described previously. Both Efron's estimation approach and the proposed one will be applied for comparison. These approaches will be further compared using simulations.

### Analyzing 2D Gel Images

At first, we analyze the *z_i _*values for the treatment, gender, and interaction effects using Efron's *locfdr *R package. The upper panel of Figure 2 shows the histograms (50 bins) of the corresponding *z*-values, the mixture density from the Poisson regression, and the null component estimated using CME and MLE. For estimation of the null component, we chose the intervals [-1.25, 0.25], [-2.5, -1.2] and [-0.5, 1.2] for the treatment, gender and interaction effects, respectively. The degrees of freedom of the splines were chosen to minimize the AIC criterion [33], which were 5, 10 and 10 respectively. The green solid curves in the upper panel of Figure 2 are estimates of the mixture densities from the Poisson regression. The blue dashed and red dotted curves in the upper panel represent the empirical null component estimated using the CME and MLE methods, respectively. The lower panel of Figure 2 shows the local FDR at different *z*-values based on the empirical null component from the CME (blue solid line) and MLE (red dotted line) methods. Figure 2 clearly conveys the message that the theoretical null, the standard normal density *N*(0, 1), is not appropriate for the proteomic data at hand. Taking the treatment effect as an example, the empirical null distribution is *N*(-0.595, 0.915^2^) by CME and *N*(-0.48, 0.891^2^) by MLE with proportions of true null hypotheses close to 1 for both, which indicates nicotine exposure effect affects similarly all proteins expressed by spleen cells. Clearly, the empirical null density is even further from its theoretical form for the gender effect. The central peak of the *z*-values is to the left of -1.

**Figure 3:**
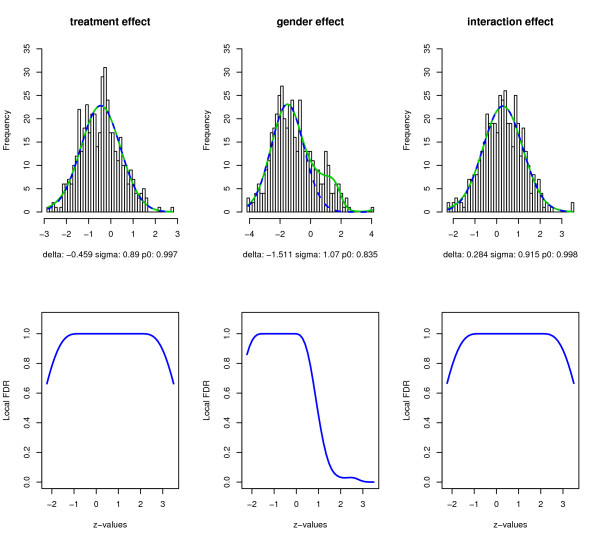
**Estimation results using the constrained approach**. Estimates of mixture densities and their null components from the constrained estimation approach and the corresponding local FDR estimation. Upper panel: the solid green curves are the spline-fitted mixture densities; the blue dashed curves are the empirical null densities from constrained estimation approach. Lower panel: the blue solid curves represent the local FDR estimates.

Figure 2 also demonstrates that neither CME nor MLE yields a desirable empirical null estimate. The estimated null components are not below the estimated mixture density throughout the range of *z*-values. Consequently, the estimated local FDR has multiple peaks and values greater than 1 at many *z*'*s*. The estimate for the proportion of true null hypotheses can also be greater than 1, which is not a desirable outcome. There is significant discrepancy between the results from CME and MLE, as demonstrated by plots for the gender effect. We tried alternative specifications for the intervals used for estimating the empirical null density and different degrees of freedom for the splines: all yielded very similar results. Moreover, we found that MLE is more sensitive to the choice of the interval [*a*, *b*] as also observed in [24]. Next, we applied the proposed constraint estimation approach with the same choices of null intervals. The degrees for the splines that minimize the AIC were 5, 9 and 5 for the treatment, gender and interaction effects, respectively. Figure 3 displays the results. The green solid and blue dashed curves in the upper panel represent the mixture and empirical null densities, respectively. The lower panel shows the estimated local FDR at different *z*-values.

Comparing with Figure 2, we see that the proposed constrained estimation approach yielded results similar to those obtained with CME. However, now, the empirical null component is below the mixture density, and the local FDR estimate is no greater than 1, smooth and non-increasing at both tails. For treatment and interaction effects, the null proportion is nearly one, indicating that there is no apparent differential effect of nicotine exposure. The treatment and interaction effects follow approximately *N*(-0.459, 0.89^2^) and *N*(0.284, 0.915^2^), respectively. The empirical null distribution for the gender effect s *N*(-1.511, 1.07^2^) with the null proportion about 0.84. The results for the gender effect show that we need to interpret results from large-scale hypothesis testing with caution. The bulk of the histogram is centered around -1.5, indicating that the majority of proteins have higher expression in female rats. The local FDR plot for the gender effect reveals that there is a small group of proteins with higher expression in males. This group of proteins is clearly separate from the rest as evidenced by the small local FDR. The local FDR is therefore more indicative of how different the gender effect is on a protein compared to the majority of the proteome, and less indicative of how significant the gender effect is. Should the theoretical null distribution be used, there would be a large number of effects at the left tail. Overall, we note that the estimated means of the null components are far from zero, especially for the gender effect, which may indicate the need to further normalize the data to remove some systematic bias.

### Simulation Validations

#### Numerical simulation

**Figure 4:**
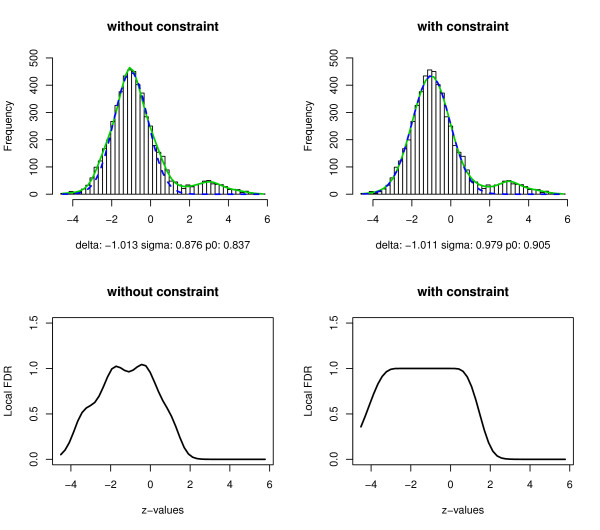
**Estimation results for simulated z-values**. Histogram of simulated *z*-values, estimated mixture densities (green solid curves) and null components (blue dashed curves) using the CME approach (left column) and the constrained estimation approach (right column). The lower panel displays the estimated local FDR for each approach.

In this section, we compare the proposed constrained estimation procedure with the CME approach without constraint using numerical simulations. The simulation model consists of *z_i _*~ *N*(-1, 1), *i *= 1, ..., 5000, and *z_i _*~ *N*(3, 1), *i *= 5001, ..., 5500. Thus, the first 5000 $z_{i}^{\prime }\text{s}$ belong to the null distribution and the last 500 $z_{i}^{\prime }\text{s}$ to the non-null distribution, and the null proportion *p*_0 _= 0.909. The interval [-2, 0] was used for estimating the null component. The estimated mixture density and its null component are displayed in Figure 4, with the left column showing the results from the CME approach without constraint and the right column showing the results from the proposed constrained estimation approach. The upper panel shows the histogram of the simulated *z*-values from one run, the estimated mixture density (solid green curve) and the empirical null component (blue dashed curve). The lower panel shows the estimated local FDR from each approach.

Even when the true null distribution is normal and there is a large number of observations, the unconstrained estimation approach generated undesirable results. The null component is greater than the mixture distribution at some points around the peak of the histogram. Moreover, the left tail of the local FDR is close to 0, indicating that some true null values will be declared as non-null depending on the threshold of the local FDR. The estimated null density follows *N*(-1.013, 0.876^2^) with the null proportion ${\hat{p}}_{0}=0.837$, which is quite different from the values in the simulation model. In contrast, the empirical null density estimated using the constrained estimation approach is more accurate. The estimated empirical null density follows *N*(-1.011, 0.979^2^) with ${\hat{p}}_{0}=0.905$. The right tails of the estimated local FDR are similar under the two approaches, which indicates that both have similar sensitivity. The left tail of the local FDR has much larger values in the constrained method, indicating a lower chance of a true null value being declared as a non-null.

We performed 100 simulations to compare the bias and standard deviation of estimates of the null parameters from both approaches. We chose different numbers of bins (50 bins or 100 bins) as well as different numbers of observations (N = 550 or N = 5500). Table 1 shows the mean and standard deviations (SD) of the estimates of the null parameters from both approaches.

**Table 1:** Comparison of Estimates for Null Parameters (*δ *= -1, *σ *= 1, *p*_0 _= 0.909; 100 simulations).

50 bins, *N *= 550			100 bins, *N *= 550		100 bins, *N *= 5500	
**mean, SD**	**unconstrained**	**constrained**	**unconstrained**	**constrained**	**unconstrained**	**constrained**

*δ*	-1.008	-1.001	-1.002	-0.995	-0.999	-1.000
SD	0.089	0.056	0.097	0.058	0.032	0.020

*σ*	0.997	0.992	1.000	0.991	1.004	0.994
SD	0.164	0.043	0.125	0.043	0.045	0.017

*p*_0_	0.914	0.905	0.916	0.906	0.913	0.907
SD	0.108	0.011	0.076	0.012	0.025	0.005

From Table 1, we see that both approaches yielded estimates that are nearly unbiased. The estimates from the proposed approach have much smaller standard error, especially for *σ *and *p*_0_. The superior performance of the constrained procedure continues as the total number of observation increases. The constrained approach is not sensitive to the number of bins used for estimation when this number is large enough (50 or 100) for the histogram counts to be roughly proportional to the density in the bins. The unconstrained approach is more affected by the number of bins, with a smaller number leading to increased variability for the estimates of *σ *and *p*_0_. The simulation results clearly demonstrate that the constrained approach is better at estimating the null component.

Next, we compare the performance of both approaches for estimation of the local FDR at points close to the non-null component. To do that, we choose several *z*'s on the right tail to compare the local FDR estimates with the true values. The results are shown in Table 2. The comparison is based on the ratio of the average of the local FDR estimates at a given *z *to the true value and on the relative SD of the estimates from the two approaches for the 100 simulations. The relative SD was computed as the SD from the constrained approach divided by the SD from the unconstrained approach.

**Table 2:** Comparison of Estimates for Local FDR (100 simulations).

		50 bins, *N *= 550		100 bins, *N *= 550		100 bins, *N *= 5500	
***z***		**unconstrained**	**constrained**	**unconstrained**	**constrained**	**unconstrained**	**constrained**

2	ratio	1.42	1.04	3.80	1.20	1.17	1.00
	relative SD		0.24		0.05		0.40

2.5	ratio	1.99	1.17	1.25	1.30	1.30	1.00
	relative SD		0.23		0.02		0.25

3	ratio	3.16	1.14	62.0	1.30	1.48	0.95
	relative SD		0.14		0.003		0.15

3.5	ratio	8.98	1.17	535.6	1.30	1.94	0.97
	relative SD		0.04		0.0004		0.10

4	ratio	35.9	1.34	764.6	1.60	2.81	1.00
	relative SD		0.01		0.00004		0.06

Table 2 clearly shows that the estimate of the local FDR from the proposed procedure has smaller bias, much less variability, and converges to the true value faster when *N *increases. The bias (relative to the magnitude of the true values) in the unconstrained approach increases with greater values of *z *(smaller local FDR), and larger number of bins when *N *is fixed. The bias of both approaches decreases when *N *increases. When *N *is not so large and the number of observation per bin is small, the unconstrained approach leads to much larger variability and bias for smaller true local FDR values. Overall, the performance of constrained estimation is much more stable and not sensitive to the number of bins as well as to the magnitude of the true local FDR values.

#### Validation using Simulated Gels

**Figure 5:**
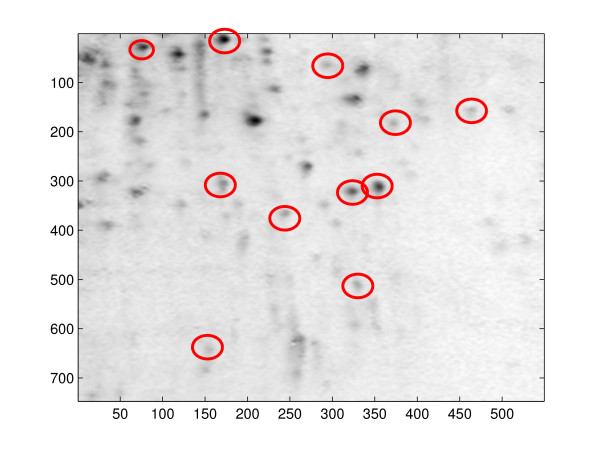
**A simulated gel image from group 1**. A simulated gel image from group 1. The 11 altered spots are circled.

**Figure 6:**
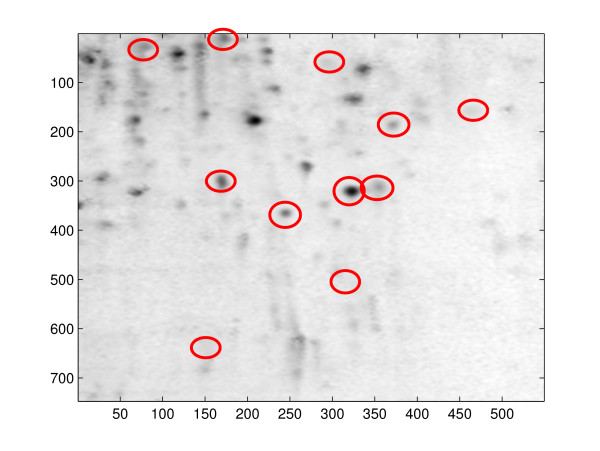
**A simulated gel image from group 2**. A simulated gel image from group 2. The 11 altered spots are circled.

**Figure 7:**
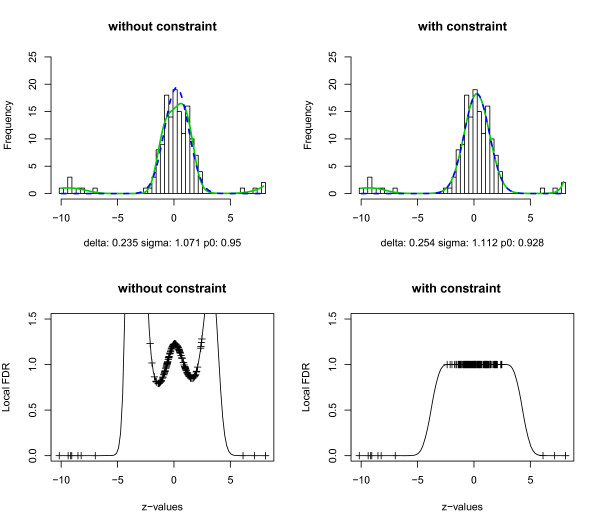
**Estimation results for simulated 2D gel images**. Histograms of *z*-values from simulated gels, estimated mixture densities (green solid curves) and null components (blue dashed curves) using the CME approach (left column) and the constrained estimation approach (right column). The lower panel displays the estimated local FDR for each approach. The '+' signs in the lower panel locate the observed points.

To further validate the proposed approach, we analyzed a set of simulated 2D gel images, which was generated by randomly perturbing an actual gel image as described in [29]. The 20 simulated gels were divided into two groups of 10. To simulate the group (treatment or intervention) effect, we artificially altered 11 manually selected spots such that these 11 spots were significantly differentially expressed across groups. Figures 5 and 6 show two simulated gel images from different groups with the 11 altered spots circled. The test statistics for the 147 spots were obtained using the RegStatGel software. We applied both estimation approaches. The results are shown in Figure 7. The interval [-2.5, 2.5] was used for estimating the null component. The left column shows the results from the CME approach without constraint and the right column shows the results from the proposed constrained approach. The upper panel shows the histogram of *z *values, the estimated mixture density (solid green curve) and the empirical null (blue dashed curve). The lower panel shows the estimated local FDR from each approach. The '+' signs in the lower panel locate the observed points. Both approaches identified all and only the 11 spots. Both approaches yield local FDR estimates for the 11 spots much lower than for the other proteins. Again, the unconstrained approach shows a bizarre local FDR curve.

## Conclusions

Similar to microarray data analysis, proteomic analysis leads to large-scale simultaneous hypothesis testing and thus carries similar challenges. The two-component model plays an important role in the microarray literature. We applied a two-component EB model for analyzing a set of 2D gel images. As demonstrated by the 2D gel data, the true null density can be very different from its theoretical form, which supports Efron's innovative idea of choosing the empirical null distribution for hypothesis testing. The problem of estimating the null density is important and fundamental in the two-component EB model. Efron generalized the theoretical null *N*(0, 1) to *N*(*δ*, *σ*^2^) and proposed two methods, CME and MLE, for estimating the null density, which are convenient to use.

However, as shown here, neither method is devoid of problematic results, which are hard to interpret in practice. To improve the estimation of the null density, we proposed a constrained estimation approach based on the central matching method. This novel procedure naturally takes the shape of the null density and its relationship to the mixture density into account for estimation, and explicitly constrains the estimated mixture density to being no less than the null density. Both the unconstrained and constrained approaches are nearly unbiased. The constrained method yields more stable and desirable estimation, as demonstrated by our simulation results. It can be generalized to include the situation where the null density comes from a family broader than the normal. The proposed approach can certainly be applied to any context where large-scale hypothesis testing occurs. Here, we have constrained the null component to be no greater than the mixture density for the histogram bins. It is a simplified version of the constraint that the null component is no greater than the mixture density over the entire real line, which is much more complicated. We note that, given the smoothness of the mixture density, the simplified constraint suffices in practice. It is reasonable to assume that the local FDR is a non-increasing function near the tail areas where the *z*-values are farther away from the null component. To impose this non-increasing property on the estimation of the local FDR, the monotone spline regression technique [34] should be utilized. We will tackle this in our future work.

The choice of the interval [*a*, *b*] may be influential for the estimation, especially if it is misspecified. When it is appropriately specified, i.e., the non-null component is nearly zero in the interval, our limited experience showed that the proposed approach is not sensitive to the choice of [*a*, *b*]. However, how the interval [*a*, *b*] can affect the estimation in general needs further research.

A quite different method for empirical null estimation is based on Fourier analysis [35]. Rather than modeling the mixture density, an attractive method for modeling the local FDR directly has also been proposed [25]. The former is non-parametric and the latter relies on parametric model assumptions. Both methods yield good estimates.

We have focused on estimating the local FDR based on test statistics. The two-component EB model is robust to correlation effects among the test statistics. It may be more informative to model the structure inherent in the data, which is certainly a challenging problem and relies on model assumptions. Further research is certainly needed here.

We utilized the protein quantifications from software RegStatGel with default settings. It should be noted that different software may generate different quantifications [36]. It is beyond the scope of the current paper to compare different quantifications.

## Authors' contributions

FL developed the constrained estimation approach and generated all the numerical results, as part of his doctoral work. FSM and FL wrote the paper together. All authors read and approved the final manuscript.
